# Spatial Learning Is Associated with Antagonist Outcomes for DNA Methylation and DNA Hydroxymethylation in the Transcriptional Regulation of the Ryanodine Receptor 3

**DOI:** 10.1155/2021/9930962

**Published:** 2021-08-11

**Authors:** Rodrigo F. Torres, Bredford Kerr

**Affiliations:** ^1^Departamento de Ciencias Básicas, Facultad de Medicina, Universidad Católica de la Santísima Concepción, Concepción 4090541, Chile; ^2^Centro de Estudios Científicos, Valdivia 5110466, Chile; ^3^Centro de Biología Celular y Biomedicina-CEBICEM, Facultad de Medicina y Ciencia, Universidad San Sebastián, Santiago 7510157, Chile

## Abstract

Increasing attention has been drawn to the role that intracellular calcium stores play in neuronal function. Ryr3 is an intracellular calcium channel that contributes to hippocampal long-term potentiation, dendritic spine function, and higher cognitive processes. Interestingly, stimuli that increase neuronal activity upregulate the transcriptional activity of Ryr3 and augment DNA methylation in its proximal promoter. However, if these observations are valid for complex behavioral tasks such as learning and memory remains being evaluated. Relative expression analysis revealed that spatial learning increased the hippocampal levels of Ryr3, whereas mice trained using a visible platform that resulted in no spatial association showed reduced expression. Interestingly, we also observed that specific DNA modifications accompanied these opposite transcriptional changes. Increased DNA methylation was observed in hippocampal samples from spatially trained mice, and increased DNA hydroxymethylation was found in samples from mice trained using a visible platform. Both DNA modifications were not altered in control regions, suggesting that these changes are not generalized, but rather specific modifications associated with this calcium channel's transcriptional regulation. Our two experimental groups underwent the same physical task differing only in the spatial learning component, highlighting the tight relationship between DNA modifications and transcriptional activity in a relevant context such as behavioral training. Our results complement previous observations and suggest that DNA modifications are a reliable signal for the transcriptional activity of *Ryr3* and can be useful to understand how conditions such as aging and neuropathological diseases determine altered *Ryr3* expression.

## 1. Introduction

Daily experiences that result in long-term memory formation are accompanied by cellular modifications that allow information storage [1, 2]. Dynamic gene expression is one of the mechanisms contributing to the synaptic plasticity underlying higher cognitive processes [2, 3]. DNA modifications are a mechanism contributing to the dynamism of gene expression [2, 4], and although increasing information is currently available, how these modifications relate to spatial learning behavior is a complex matter that remains to be established.

DNA methylation and DNA hydroxymethylation are highly dynamic epigenetic modifications that change not only along the lifetime [5, 6] but also in response to external stimuli [1, 4] and might underlie the development of pathological conditions [7]. These DNA modifications have been shown to act as molecular switches for gene expression [2]. For instance, DNA methylation has been generalized as a repressive modification, and conversely, DNA hydroxymethylation is considered a transcriptional activator [2]. However, our current understanding suggests that the role that DNA modifications have on gene expression is highly dependent on the chromatin context [4, 8–10].

Intracellular calcium stores play a vital role in neuronal function, contributing to processes ranging from apoptosis to memory consolidation [11, 12]. The ryanodine receptors (RyRs) are intracellular calcium channels that contribute to hippocampal synaptic plasticity and higher cognitive processes such as learning and memory [11, 13, 14]. The ryanodine receptor 3 (Ryr3) is relevant for hippocampal function and spatial memory, as Ryr3-KO mice showed altered hippocampal synaptic plasticity and impaired performance in the Morris water maze. Similarly, Ryr3 knockdown in mice resulted in altered memory in the passive avoidance test [15–18], suggesting a role for Ryr3 in neuronal function. We and others have shown that experiences that increase neuronal activity are associated with the upregulation of *Ryr3* [14, 19, 20]. Moreover, we showed that in mice reared in an enriched environment, DNA methylation and Mecp2 binding to the *Ryr3* promoter were required for its transcriptional upregulation [20], raising our interest to explore the transcriptional regulation of *Ryr3* in the context of learning and memory.

To establish if spatial learning induced a transcriptional regulation of the *Ryr3* gene, we trained mice to find a hidden platform using spatial cues. A learning control was trained using a visible platform, therefore maintaining the physical task but lacking the spatial association requirement. We observed that both groups showed opposite transcriptional responses, increasing and decreasing the transcriptional activity of *Ryr3*, respectively. To understand if this discrepancy could be associated with DNA modifications, we evaluated hippocampal DNA methylation and DNA hydroxymethylation in the *Ryr3* proximal promoter. We observed that the upregulation of *Ryr3* observed in the spatially trained mice was associated with increased DNA methylation. Contrastingly, the transcriptional depression observed for the control group was associated with increased hydroxymethylation in the *Ryr3* proximal but not in an upstream region.

Our observations show an antagonist role for DNA methylation and DNA hydroxymethylation in the hippocampus of mice that underwent a behavioral training that resulted in long-term spatial memory compared to those who did not develop a spatial association, highlighting the relevance of DNA modifications in directing gene activity when contextualized to higher cognitive processes such as learning and memory.

## 2. Material and Methods

### 2.1. Mice

C57BL/6J mice were used and kept under 12 h-12 h light-dark cycles. Food and water were provided *ad libitum.* Experiments were developed according to the guidelines and regulations emitted by the Fondo Nacional de Desarrollo Científico y Tecnológico (FONDECYT) and were approved by the Centro de Estudios Científicos Animal Care and Use Committee. The mouse facility of the Centro de Estudios Científicos (CECs) is accredited by the Association for the Assessment and Accreditation of Laboratory Animal Care International (AAALAC). All methods were performed in accordance with the ARRIVE guidelines and regulations.

### 2.2. Morris Water Maze and Experimental Groups

The Morris water maze (MWM) was performed as previously described [20]. Briefly, the pool (120 cm diameter) was filled with water (22-24°C) until it reached a 50 cm deep. Nontoxic white paint was used to opaque the water, and spatial cues surrounded the pool at a 1 m height. Each training day considered four 60-second trials. The mice began each trial from random points of the pool. All sessions were video-recorded, and escape latency was registered. Remote memory was evaluated in a single trial session that occurred 21 days after the spatial training. Time spent in the target and opposite quadrants of the pool were measured. Mice used in this study were euthanized 4 hours after the last day of training.

Mice were randomly divided into three experimental groups named hidden, visible, and home-cage. The hidden group: mice were trained for six consecutive days using an escape platform kept hidden and in a fixed position relative to the spatial cues through all sessions. The visible group: this learning-control group was trained using a visible platform that changed its position each trial, therefore lacking a spatial association. This group was trained for four consecutive days. All trials were performed at the same hour of the days. Although both groups were trained for different periods of time, six days for the hidden and four days for the visible groups, this difference allowed us to obtain the maximal behavioral similarity between groups. After the behavioral training, all processing times were identical for mice in all the conditions. The home-cage group: this group of mice was not trained and was kept in the home-cage throughout the study. All mice were euthanized 4 hours after the last training trial, and in the case of home-cage group, they were euthanized at the same hour of the day with the mice from the other two groups.

For remote memory evaluation, randomly selected mice from each behavioral condition were kept alive for 21 days after training and subjected to a single trial spatial memory evaluation. Swim trajectories were obtained using the Bonsai visual reactive programming software (https://bonsai-rx.org/). These mice were euthanized 4 hours after this evaluation. Whole hippocampus was recovered from sagittal sectioning of the hemispheres and used as substrate for the molecular analyses.

### 2.3. DNA Methylation and DNA Hydroxymethylation

Relative DNA methylation levels were estimated using an MBD-based enrichment of methylated DNA, followed by qPCR [21]. The MethylMiner Methylated DNA Enrichment Kit (ME10025, Invitrogen) was used according to the manufacturer's instructions. Briefly, DNA was sonicated to obtain fragments of an average of 250 bp. Methylated DNA from whole hippocampus was captured with a biotin-conjugated methyl-CpG-binding domain from MBD2 in magnetic beads coated with streptavidin. Captured DNA was eluted with buffers containing increasing salt concentrations, eluting low methylated DNA in the first fraction, and high methylated DNA in the last fraction [21]. These fractions were used as a substrate for qPCR using primers encompassing the proximal promoter of the *Ryr3* gene and the H19 locus [22]. Abundances of the region of interest were determined for each behavioral condition and the low and high methylated fractions, using the following primer pair located in the *Ryr3* proximal promoter F: TGCATAGAGCAAACGCAGGT and R: AGAGCATGCCTAAGTGGTCG. Percentage of enrichment was determined in relation to the home-cage group. Artificial fragments of methylated and nonmethylated DNA were incorporated into each experiment to control each procedure. DNA hydroxymethylation was measured using the EpiJET 5-hmC Enrichment Kit (#K1491BID, Thermo Scientific) according to manufacturer instructions. This method ensures the capturation of hydroxymethylated DNA by adding an enzyme to modify the 5-hme into a biotin linker. Hence, avoiding any possible DNA methylation cross reactivity, DNA was sonicated to fragments averaging 250 bp. The fragmented DNA from whole hippocampus were incubated with the modifying enzyme and subjected to biotinylation. Magnetic beads containing streptavidin were used to capture the modified DNA. Control fragments of hydroxymethylated and nonhydroxymethylated DNA were used to test the enrichment procedure. The samples enriched in hydroxymethylated DNA were subjected to qPCR analysis using the set of primers of interest. Procedures for DNA methylation and DNA hydroxymethylation analyses were performed using 1 *μ*g hippocampal DNA from each mouse as substrate. The percentage of enrichment was obtained in relation to the home-cage group.

### 2.4. Gene Expression

RNA from whole hippocampus was isolated and reverse transcribed as previously described [23]. Briefly, brains were dissected, and samples were homogenized in Trizol according to manufacturer's instructions. RNA was precipitated and treated with one unit of DNase I (Life Technologies). Five micrograms of total RNA were reverse transcribed using random primers and ImProm II kit (Promega). cDNA was quantified by qPCR using Kapa SYBR Quantimix (Kapa). The qPCR analysis was performed in triplicates from one reverse transcribed product using the Rotor-Gene 6000 (Corbett). Values were analyzed following the 2^−ΔΔCt^ method using cyclophilin-A (Cyc) and *β*2-microglobulin (B2m) as normalization controls using the following primer pairs: Ryr3 F:TGGTGTCGGTGATGATCTGT and R:TGCACAGGTTGTCCATTGAT [4, 20]; Cyc F:GGCAATGCTGGACCAAACACAA and R:GTAAAATGCCCGCAAGTCAAAAG; B2m F:GCTATCCAGAAAACCCCTCAA and R:CATGTCTCGATCCCAGTAGACGGT [23, 24].

## 3. Results

**Figure 1:**
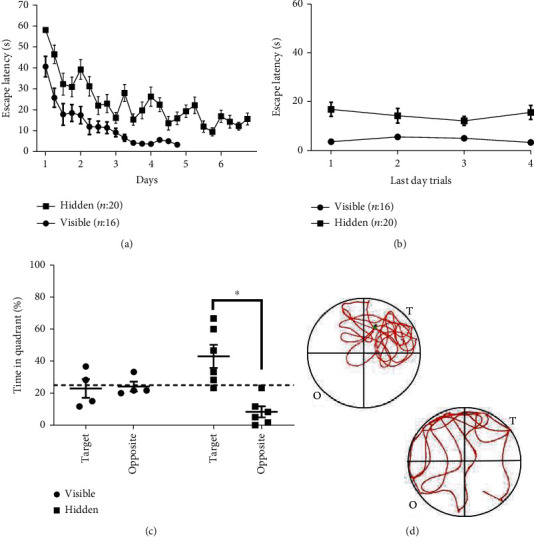
Training in the Morris water maze. (a) Training course: escape latency is shown for a group of mice trained to find a hidden platform for six days and a control group trained for four days using a visible platform. Each day consisted of 4 trials to complete a total of 16 trials (visible) and 24 trials (hidden). (b) Escape latencies obtained for the four trials of the last day of training for both groups of mice. (c) Remote memory evaluated as the time spent in the target vs. opposite quadrant of the water maze during a single trial session performed 21 days after training for each behavioral group. The time spent in the target and opposite quadrant from each mouse was evaluated. The visible (circles) and hidden (squares) groups were analyzed independently. Dashed line shows the expected preference for a random swim trajectory (paired *T*-test, *p* = 0.081). Graphs show individual values and mean ± SEM. (d) Representative swim trajectories for mice trained using a hidden (upper) and visible (lower) platform; the target (T) and opposite (O) quadrants are shown.

To generate a long-lasting spatial memory, we trained mice in the MWM using a hidden platform for six consecutive days (hidden group). A learning-control group was trained for four days using a visible platform that changed location each trial (visible group). This group allowed us to monitor modifications that could arise as a consequence of aspects other than spatial learning, such as the swimming exercise and daily handling. The training course showed that the visible group reached a trial-to-trial consistency faster than the hidden group (Figures 1(a) and 1(b)). However, we observed that the last four trials of the hidden group resulted in escape latencies averaging below 15 seconds, irrespective of the trial-start position, suggesting that spatial learning was achieved (Figure 1(b)). To determine if our six days of training resulted in a long-lasting memory, randomly selected mice from each behavioral condition were evaluated for remote memory. The time spent in the target (previous location of the platform) and opposite quadrant was registered for each mouse during single one-trial session performed 21 days after the MWM training. We observed that the visible group developed no spatial association with any quadrant (Figures 1(c) and 1(d)). Contrastingly, the hidden platform group spent significantly more time in the target than in the opposite quadrant of the pool (Figure 1(c), paired *T*-test, *p* = 0.0181, Figure 1(d)), suggesting that our training resulted in a long-lasting spatial memory.

**Figure 2:**
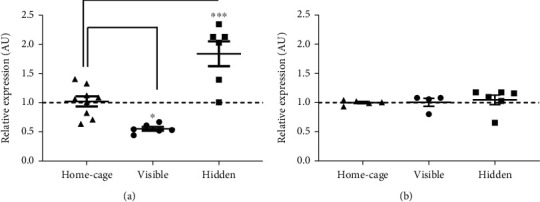
*Ryr3* gene expression in different behavioral conditions. (a) Hippocampal posttraining *Ryr3* gene expression evaluated for the visible and hidden groups in relation to mice kept in the home-cage. Gene expression of the hidden and visible groups were compared to that of the home-cage group using ANOVA with Bonferroni's post hoc test, *p* = 0.0266 and 0.0003 for the visible and hidden groups, respectively. (b) Hippocampal *Ryr3* gene expression evaluated 21 days after training in the remote spatial-memory session for the visible and hidden groups in relation to mice kept in the home-cage. Graphs show individual values, means ± SEM. Basal gene expression for the home-cage group is shown as a dashed line.

To determine whether *Ryr3* transcriptional regulation was involved in the long-lasting spatial memory, we then evaluated the transcriptional activity of the *Ryr3* gene. As expected, spatial learning in the MWM resulted in an upregulation of Ryr3 mRNA when contrasted to the home-cage group (Figure 2(a), ANOVA, *p* = 0.0003). However, to our surprise, the control group trained using a visible platform showed diminished *Ryr3* mRNA levels compared to the home-cage group (Figure 2(a), ANOVA, *p* = 0.0266). This data indicates that the same physical task performed with or without the spatial-learning component resulted in opposite transcriptional regulation of this gene. *Ryr3* relative expression evaluated in mice subjected to the remote spatial memory evaluation revealed no differences for the different behavioral conditions (Figure 2(b)). These data suggest that the hippocampal transcriptional regulation of *Ryr3* is transient and specifically induced during spatial learning, but not in a remote memory evaluation.

**Figure 3:**
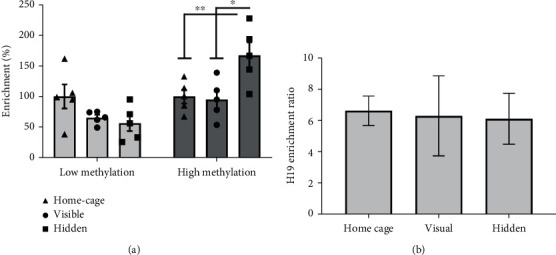
DNA methylation changes induced by behavioral training in the Morris water maze. (a) Methylated DNA percentage of enrichment for the hidden and visible platform groups in relation to the home-cage group evaluated for the *Ryr3* proximal promoter. Results are shown for the elution obtained with a low-salt buffer (low methylation) and high-salt buffer (high methylation). The abundance of the Ryr3 promoter was evaluated in each elution and compared between conditions (2-way ANOVA with Tukey's post hoc test, *p* = 0.0122 and 0.0070 for the home-cage and visual comparison, respectively). (b) Enrichment of methylated DNA for the H19 locus shown as a High methylated/low methylated DNA ratio for each behavioral condition. Graphs show individual values and mean ± SEM.

Considering our previous observations relating DNA methylation to the transcriptional activity of the *Ryr3* gene, we hypothesized that a change in DNA methylation in the proximal promoter of this gene could underlie the transcriptional regulation with opposite valence observed for the hidden and visible groups. To this end, we used a biotin-MBD protein conjugate to capture methylated DNA from hippocampal samples of mice euthanized after the last training session from every behavioral condition. This method allowed us to obtain a low-methylated and a high-methylated fraction for each sample. We evaluated these fractions for the abundance of the Ryr3 proximal promoter and the H19 locus an imprinted and highly methylated region as control. The low-methylated fraction did not reveal any differences between conditions. Contrastingly, the high-methylated fraction showed increased abundance of the *Ryr3* proximal promoter only in the group of mice trained with the hidden platform, in which a spatial memory was acquired (Figure 3(a), 2-way ANOVA, *p* = 0.0122 and 0.0070 for the hidden/home-cage and hidden/visual comparisons, respectively). To determine the enrichment protocol's effectiveness, we performed the analysis for the H19 locus beside procedural controls. The high-methylated/low-methylated ratio for the H19 locus showed that our enrichment protocol resulted in a 6-fold greater representation for a known methylated region in the high-methylated over the low-methylated fraction. This suggests that our enrichment protocol was effective and similar for all behavioral conditions (Figure 3(b)). These observations show that the upregulation of *Ryr3* is associated with increased DNA methylation levels in its proximal promoter. More interestingly, this data also suggests that the transcriptional depression observed for the visible group was not due to decreased DNA methylation in the *Ryr3* proximal promoter.

**Figure 4:**
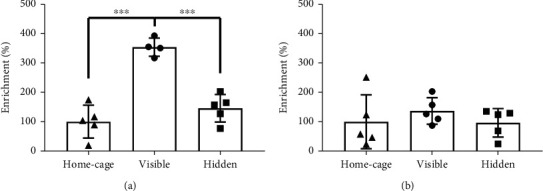
DNA hydroxymethylation change measured for the *Ryr3* proximal promoter and a control distal region of the gene. (a) Percentage of enrichment for DNA hydroxymethylation for the hidden and visible platform groups in relation to the home-cage group evaluated in the Ryr3 proximal promoter. The abundance of the Ryr3 gene promoter was evaluated and contrasted between groups (one-way ANOVA with Bonferroni's post hoc test *p* < 0.0001 and 0.001 for home-cage and hidden comparison, respectively). (b) Percentage of enrichment of DNA hydroxymethylation for the hidden and visible platform groups in relation to the home-cage group evaluated in a *Ryr3* distal upstream region. Graphs show individual values and mean ± SEM.

To understand if the transcriptional depression observed in the group of mice trained with a visible platform could be associated with a DNA modification, we turned our attention to DNA hydroxymethylation. To this end, we evaluated the levels of hydroxymethylated DNA in the proximal promoter of *Ryr3* in hippocampal samples of mice from the different behavioral conditions. Interestingly, we observed an enrichment of hydroxymethylated DNA in the proximal promoter of the *Ryr3* gene only for the learning-control group of mice (Figure 4(a), one-way ANOVA, *p* < 0.0001 and 0.001 for home-cage/visible and hidden/visible comparisons, respectively). To determine if the increase in hydroxymethylated DNA observed in mice trained using a visible platform was a promoter-specific effect, we evaluated a distal region upstream of the *Ryr3* gene. We observed no differences in the levels of hydroxymethylated DNA among the different behavioral conditions for this location (Figure 4(b)), suggesting that our observation is not a generalized effect of the behavioral condition but rather a promoter-specific epigenetic change associated with the transcriptional regulation of *Ryr3*.

## 4. Discussion

Understanding learning and memory as a cellular process has led to several outstanding discoveries and remains an intriguing and passionate field. Here, we used the Morris water maze to interiorize into the role of DNA modifications in directing transcriptional activity in the context of learning and memory using the *Ryr3* gene as a model. Our training paradigm resulted in a robust spatial memory, evident even 21 days after training. To ensure that our observations were associated with spatial learning, we used a control that was trained using a visible platform that changed location each trial. Hence, this group required no spatial association to reach the platform. Although these learning-control mice were trained for fewer days than the hidden group. The behavioral similarity between groups was maximized. Moreover, this learning-control underwent the same handling, stress, and physical exercise than the spatial learning group.

Both groups showed antagonist transcriptional responses in the hippocampus. It was unexpected but useful for understanding the transcriptional regulation within the broader context of learning and memory. Our observation that the transcriptional upregulation of *Ryr3* is associated with increased DNA methylation in this spatial learning paradigm is consistent with previous data obtained from the hippocampus of mice subjected to electroconvulsive shock or reared in an enriched environment [4, 20], suggesting that DNA methylation is a reliable signal for increased transcriptional activity of the *Ryr3* gene. Interestingly, we previously showed that mice trained using a hidden platform for 4 days show increased RyR3 levels [20], suggesting that the transcriptional upregulation of Ryr3 is associated to spatial learning independently of the training period. On the other hand, knockdown of *Ryr3* has been shown to impair memory in the passive avoidance test [16], raising interest in the transcriptional depression observed for the group of mice trained with a visible platform. Further experiments could be performed to determine if this decreased transcriptional activity is behaviorally relevant to avoid the generation of a spatial association. Particularly, different hippocampal regions can be engaged and show differential transcriptional regulations, as dorsal hippocampus has been extensively associated to spatial learning and ventral hippocampus to fear behavior or anxiety [25]. It is also possible that different neuronal populations were differentially engaged in the spatial-learning and learning-control groups leading to the opposite responses and DNA modifications, as it has been observed elsewhere [26]. Interestingly, the downregulation of *Ryr3* observed for the visible group was not associated with reduced DNA methylation but rather with increased DNA hydroxymethylation in the proximal promoter. A plausible scenario is to consider DNA hydroxymethylation as functional demethylation, as previously suggested [27]. This consideration is interesting as proposed that the effect of DNA hydroxymethylation on Mecp2-target genes relies in modifying the association of Mecp2 to promoters [27], and we have previously shown that the lack of Mecp2 results in *Ryr3* downregulation as well as Mecp2 binding to Ryr3 promoter is associated to its transcriptional upregulation [20]. It is also relevant to consider that given the nature of the technique used to capture methylated DNA, it is possible that non-CpG methylation was reduced but not detected in our analysis. Despite this consideration, the contrasting DNA modifications associated with differential transcriptional responses highlights the dynamism and specificity of these modifications. It is also interesting that remote-memory evaluation did not elicit a transcriptional response in any of the groups. This is consistent with the role the hippocampus play in spatial memory acquisition and consolidation [28]. Further experiments involving the prefrontal cortex could show if similar transcriptional regulation is induced by memory retrieval [28].

These modifications affecting the transcriptional activity of *Ryr3* are relevant not only in the context of learning and memory, as dysregulation of *Ryr3* has been observed in aging [29, 30], obsessive-compulsive disorder [31], and Alzheimer's disease [32, 33]. Interestingly, a biphasic effect for *Ryr3* during the early and late stages of Alzheimer's disease is proposed [34], calling attention to gain a more in-depth insight into the transcriptional regulation of the *Ryr3* gene in this context. Our observations relate two DNA modifications to antagonist outcomes in the transcriptional regulation of this gene. More importantly, these antagonist roles are specifically induced by a behavioral training that generates a long-lasting spatial memory and a training that lacks the spatial-component, establishing a relation between behavior, DNA modification, and transcriptional regulation.

## 5. Conclusion

Our observations revealed that a complex task requiring spatial-learning and a matching behavior requiring no spatial association elicited opposite transcriptional regulations of the Ryr3 calcium channel. Both conditions were associated with specific DNA modifications, suggesting that these modifications are strongly associated with the transcriptional activity of this gene and specifically induced by spatial learning. Previous observations have placed *Ryr3* as a relevant target, contributing to synaptic plasticity, dendritic spine density and learning, and memory. Understanding the mechanism directing *Ryr3* transcriptional activity in the context of behavioral training enlightens the path to understand the dynamic gene expression that underlies such relevant processes.

## Data Availability

All data generated or analyzed during this study are included in this published article.
